# The relationship between the knowledge of diabetes mellitus and the mental, psychological and emotional status of T2DM patients based on a structural equation model

**DOI:** 10.1038/s41598-022-25211-4

**Published:** 2022-12-01

**Authors:** Haiyang Li, Liping Wang, Jin Huang, Bei Li, Tieying Qiu

**Affiliations:** Clinical Nursing Teaching and Research Section, The Second Xiangya Hospital of Central South University, Changsha, 410011 China

**Keywords:** Diseases, Health care

## Abstract

To explore diabetes-related knowledge levels among patients with type 2 diabetes mellitus (T2DM) and the influencing factors, correlations and paths of patients’ mental and emotional status based on a structural equation model. A total of 1512 patients with T2DM in 18 tertiary general hospitals in Hunan Province. A descriptive correlational study. The study was conducted and surveyed with the general information questionnaires, including the Audit of Diabetes Knowledge (AD knowl), the Problem Areas in Diabetes 5 scale (PAID-5) and the World Health Organization Five-Item Well-Being Index (WHO-5). The structural equation model showed that PAID-5 and WHO-5 scores (P < 0.05) were affected by diabetes self-management, medical history/treatment and knowledge. According to the model, the common risk factors affecting the mental and emotional status of diabetic patients were general demographic characteristics (less exercise time and greater economic burden), medical history/treatment of diabetes (longer course of disease, more hospitalizations due to diabetes, and more acute or chronic complications), and lower levels of diabetes-related knowledge. In addition, solitude was also a risk factor for low levels of happiness. The diabetes-related knowledge levels of T2DM patients are very low, and these patients generally experience emotional disorders, which deserves close attention. The structural equation model can be used to explore the influencing factors and correlations of the psychological and emotional status of diabetic patients.

## Introduction

Diabetes mellitus (DM) is a chronic disease related to glucose metabolism disorder. T2DM is the most common type, accounting for 90%-95% of the total number of cases of DM^1^. In addition to the pathophysiological changes generated by DM, the related psychological, mental and emotional disorders as well as the negative changes in lifestyle caused by the need for treatment are also problems that can not be ignored in DM management. The incidence of depression in diabetic patients is higher than that in other people^2^. A large-scale cross-sectional survey of the Diabetes Attitudes, Wishes, and Needs (DAWN) study results from around the world showed that among 8596 DM patients in 17 countries, 13.8% of them may have had depression, and nearly 50% of them presented DM-related emotional distress and progressive aggravation^3,4^. In addition, approximately 40% of the survey population reported that drug treatment affected their daily lives (interpersonal communication, leisure and entertainment, work and study, economic situation, etc.) Among them, the proportion of patients on insulin reporting such problems was higher than that of patients taking only oral hypoglycaemic drugs^5^. Decreased levels of happiness and increased levels of DM stress may have negative effects on patients’ daily lives.

The purposes of this study are: (1) To understand the knowledge and psychological and emotional status of T2DM patients; (2) To examine the correlations between DM knowledge and mental, psychological and emotional status; to propose reasonable and effective suggestions for promoting physical and mental health and improving the well-being of T2DM patients; (3) To explore a new model of DM health education guided by patients’ needs.

## Materials and methods

### Study population

The subjects of this study were T2DM patients from 18 tertiary general hospitals in Hunan Province. By convenience sampling, 100 patients were selected from each hospital, including 50 T2DM patients treated with insulin and 50 T2DM patients treated with oral hypoglycaemic drugs, for a total of 1800 patients. Insulin treatment of T2DM patients refers to the use of insulin in the treatment of hypoglycaemia, which can include treatment with insulin only or treatment with both insulin and oral hypoglycaemic drugs. The inclusion criteria were as follows:

1. Meeting the 1999 World Health Organization diagnostic criteria for T2DM.
2. The age was 18 to 80 years old with the ability to listen, speak, read, and write and was not participating in other related health education at the same time.
3. Agreed to participate in the study and signed the informed consent form.
4. For the insulin treatment group, had needed to take insulin for ≥ 3 months.
5. For the oral hypoglycaemic drug treatment group, needed to take one or more hypoglycaemic drugs and had a course of disease of ≥ 1 year.

The exclusion criteria were as follows:

1. T1DM patients, gestational DM patients and other uncertain DM patients.
2. Patients with any mental disease or mental disorder.
3. DM patients with other complex diseases, such as paralysis, who could not cooperate with the researchers.

The subjects provided informed consent and participated in the study voluntarily. A total of 1800 subjects participated in the survey, and 1512 valid questionnaires were collected, with an effective rate of 84%.

### Methods

A general information questionnaire, including the AD knowl scale, the PAID-5 scale and the WHO-5 scale, were used to study the diabetes-related knowledge levels among patients with T2DM and the influencing factors, correlations and paths of patients’ mental and emotional status.

#### The AD knowl scale

The AD knowl is used to measure and evaluate an individual’s diabetes-related knowledge levels. The AD knowl includes eight dimensions with a total of 11 items: diet, treatment, sickness, foot care, physical activity, tobacco and alcohol, coping with complications, and hypoglycaemic response. Each item has three response options, i.e., “right”, “wrong”, “don’t know”; 1 point is given for correct answers, and 0 points are given for incorrect or “don’t know” answers. The higher the score is, the better the mastery of relevant knowledge. The percentage scores are divided into three levels: below 60% is poor, 60% ~ 80% is average, and above 80% is good. The second Diabetes Attitudes, Wishes and Needs study is by far the largest global research project on the psychological states, wishes and needs of diabetic patients, and the tools applied in the research have shown good reliability and validity^6^.

#### PAID-5 scale

The PAID-5 scale is used to measure and evaluate an individual’s mental state and emotional disorders. It has a total of 5 items with 5 response options, and each item is scored from 0 ~ 4. An item score of 0 points indicates that the relevant issue is “not a problem”, and a score of 4 points indicates that the issue is a “serious problem”. The total score is the sum of the individual scores multiplied by 5, and the total possible score is 100 points. The higher the score is, the more serious the emotional disorder. Total scores can be divided into two levels: 0–39 for low emotional disorder and 40–100 for severe emotional disorder.

#### WHO-5 scale

The WHO-5 scale is used to measure and evaluate an individual’s emotion or happiness. It has a total of 5 items with 6 response options, all of which are reverse scored; each item receives a score of 0 ~ 5, where 0 points means “at no time” and 5 points means “all of the time”. The raw score is calculated by totalling the five item scores and ranges from 0 to 25, with 0 representing the worst possible quality of life and 25 representing the best possible quality of life. To obtain a percentage score ranging from 0 to 100, the raw score is multiplied by 4. A percentage score of 0 represents the worst possible quality of life, whereas a score of 100 represents the best possible quality of life. The score is divided into three levels: a score ≤ 28 points indicates possible depression, a score of 29–50 points indicates decreased happiness, and a score > 50 points indicates happiness.

In addition, all methods were performed in accordance with the relevant guidelines and regulations of the Second Xiangya Hospital of Central South University.

### Construction of the structural equation model

**Figure 1:**
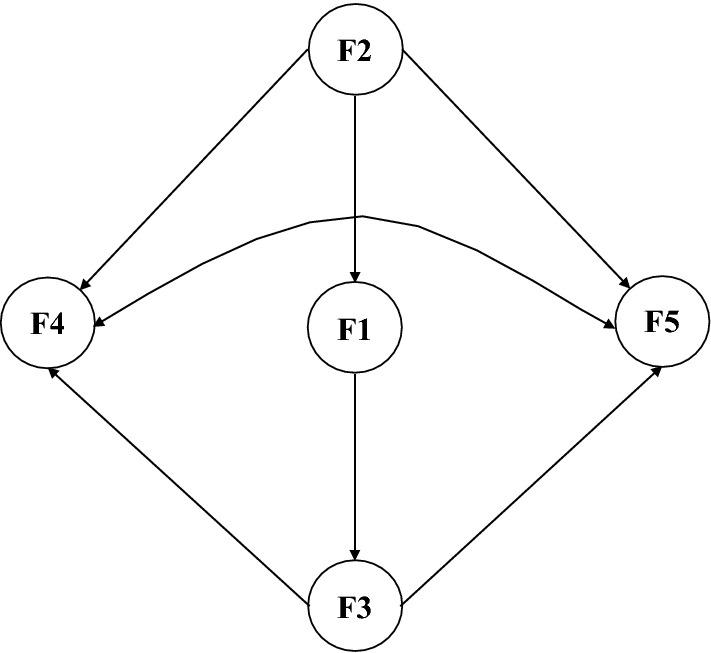
Hypothesized structural equation model of the paths of the influencing factors of problem areas in diabetes and happiness.

The study mainly discusses the influencing factors of problem areas in diabetes and well-being from four aspects: general demographic characteristics, medical history/treatment of diabetes, self-management of diabetes and diabetes knowledge. In addition to general demographic characteristics, all of the other influencing factors are latent variables (Table 1). The general characteristics examined as influencing factors of problem areas in diabetes include exercise duration, dwelling status, and the economic burden of diabetes; the general characteristics examined as influencing factors of well-being among diabetic patients include gender, marital status, exercise duration, education, dwelling status, and the economic burden of diabetes. The hypothesized path model of F1, F2 and F3 on F4 and F5 was as follows: (1) F2 → F5; (2) F1 → F5; (3) F2 → F1 → F5; (4) F2 → F1 → F3 → F5; (5) F2 → F4; F1 → F4; (6) F2 → F1 → F4; (7) F2 → F1 → F3 → F4 (Fig. 1).

**Table 1:** Influencing factors in the measurement model.

Dimension	Factors
General demographic characteristics	Gender; Marital status; Education; Dwelling status; Economic burden; Exercise duration
F1: Medical history/treatment of diabetes	A01: Duration of diabetes; A02: Number of hospitalizations for diabetes in the last year; A03: Number of chronic diabetic complications; A04: Number of acute diabetic complications
F2: Diabetes self-management	B01: Regular examinations for diabetes; B02: Regular blood glucose monitoring; B03: Established comprehensive control objectives
F3: AD knowl scale	C01: Diet; C02: Treatment; C03: Sickness; C04: Foot care; C05: Physical activity; C06: Tobacco and alcohol; C07: Coping with complications; C08: Hypoglycaemic response
F4: PAID-5 scale	D01: Feeling scared about living with diabetes; D02: Feeling depressed about living with diabetes; D03: Worrying about the future; D04: Feeling that diabetes takes up too much energy; D05: Coping with diabetes complications
F5: WHO-5 scale	E01: I have felt cheerful and in good spirits; E02: I have felt calm and relaxed E03: I have felt active and vigorous; E04: I woke up feeling fresh and rested; E05: My daily life has been filled with things that interest me

### Statistical analysis

Statistical analyses were performed using SPSS 18.0 (SPSS, Chicago, Illinois). Numerical data were statistically described as the frequency and rate (%), and measurement data were expressed as the mean ± SD. Structural equation modelling in Mplus7.0 was used to analyse the problem areas in diabetes, the influencing factors of happiness and the correlation among the influencing factors.

### Ethics approval and consent to participate

This study was approved by the Ethics Committee of the Second Xiangya Hospital of Central South University. Participants provided written informed consent to participate in this study.

## Results

### Basic information about the research objects

Among the total of 1512 subjects, 45.04% were aged from 60 to 74. There were 801 males (52.98%) and 711 females (47.02%). About 91.2% subjects were married. The most common education level was senior high school, with 29.56% of the subjects having a senior high school education. The most common occupational status was retired, with 50.79% in this category. Regarding income, 55.89% of subjects earned 1000–3000 yuan. In terms of diabetes medical expenses, 82.54% of these expenses were paid by individuals. A total of 21.03% of the subjects were smokers, and 10.98% were drinkers. In total, 67.32% of patients had DM for over 5 years, and 33.6% and 60.65% of patients had acute and chronic complications, respectively. The proportions of subjects who had regular examinations for diabetes, regular monitoring of blood glucose and established comprehensive control objectives were 37.1%, 64.17% and 42.46%, respectively (Table 2).

**Table 2:** Individuals’ baseline characteristics and basic information at follow-up.

Items	Frequency (n)	Rate (%)	Items	Frequency (n)	Rate (%)
**Age (years)**			**Exercise (mins/week)**		
≤ 44	101	6.68	< 90	575	38.03
45–59	569	37.63	≥ 90	937	61.97
60–74	681	45.04	**Dwelling status**		
≥ 75	161	10.65	Living with spouse	634	41.93
**Gender**			Living with spouse and children	734	48.54
Male	801	52.98	Living alone	90	5.95
Female	711	47.02	Other	54	3.57
**Marital status**			**Duration of diabetes (years)**		
Single/Divorced/Widowed	133	8.8	≤ 1	84	5.56
Married	1379	91.2	1.01–5	410	27.12
**Education**			5.01–10	463	30.62
Illiterate	0	0	10.01–15	266	17.59
Primary school	103	6.81	> 15	289	19.11
Junior high school	304	20.11	**Number of hospitalizations for diabetes in the last year**		
Senior high school	447	29.56	0	595	39.35
Junior college	325	21.49	1	590	39.02
Bachelor’s degree and above	188	12.43	2	177	11.71
**Occupation**			> 2	150	9.92
In-service medical officer	17	1.12	**Chronic complications**		
Active worker	137	9.06	0	595	39.35
Farmer	266	17.59	1	590	39.02
Student	9	0.6	2	177	11.71
Civil servant/teacher	119	7.87	≥ 3	150	9.92
Financial businessperson	41	2.71	**Acute complications**		
Retired	768	50.79	0	1004	66.4
Unemployed or laid off	85	5.62	1	465	30.75
Others	70	4.63	≥ 2	43	2.84
**Average personal monthly income (yuan)**			**Regular examinations for diabetes**		
< 1000	284	18.78	No	951	62.9
1000–3000	845	55.89	Yes	561	37.1
3001–8000	348	23.02	**Regular self-monitoring of blood glucose**		
> 8000	35	2.31	No	540	35.83
**Economic burden of diabetes mellitus**			Yes	967	64.17
No burden	264	17.46	**Established comprehensive control objectives**		
Some burden	858	56.75	No	870	57.54
Heavy burden	390	25.79	Yes	642	42.46
**History of smoking**			**Treatment of diabetes**		
No	1194	78.97	Oral hypoglycaemic agent therapy	492	32.54
Yes	318	21.03	Insulin therapy	454	30.03
**History of drinking**			Combined therapy	566	37.43
No	1346	89.02			
Yes	166	10.98			

### Subjects’ AD knowl scores

Among the subjects, 90.41%, 8.93% and 0.66% had knowledge scores of < 60 points, 60–79 points and ≥ 80 points, respectively. The mean total score of diabetes knowledge was 50.87 ± 13.43, with a minimum value of 0 and a maximum value of 102 (Table 3).

**Table 3:** AD knowl scores.

Items	Scores ($\overline{x}$ ± *s*)	Min	Max
Total score	50.87 ± 13.43	0	102
Diet	8.83 ± 3.23	0	19
Treatment	4.72 ± 2.37	0	15
Sickness	3.14 ± 1.90	0	8
Footcare	11.58 ± 3.63	0	24
Physical activity	2.76 ± 2.00	0	9
Tobacco and alcohol	3.88 ± 2.29	0	11
Coping with complications	7.59 ± 2.52	0	11
Hypoglycaemic response	8.37 ± 2.96	0	14

### Subjects’ PAID-5 scores

In total, 44.05% and 55.95% of subjects had PAID-5 scores from 0–39 (low emotional disorder) and 40–100 (severe emotional disorder), respectively. The mean total PAID-5 score was 43.06 ± 23.88, with a minimum value of 0 and a maximum value of 100 (Table 4).

**Table 4:** PAID-5 scores.

Items	Scores ($\overline{x}$ ± *s*)	Min	Max
Total score	43.06 ± 23.88	0	100
Feeling scared about living with diabetes	1.61 ± 1.15	0	4
Feeling depressed about living with diabetes	1.47 ± 1.11	0	4
Worrying about the future	1.96 ± 1.21	0	4
Feeling that diabetes takes up too much energy	1.62 ± 1.10	0	4
Coping with diabetes complications	1.95 ± 1.19	0	4

### Subjects’ WHO-5 scores

Among the subjects, 15.21%, 19.64% and 65.15% had WHO-5 scores of ≤ 28 points (possible depression), 29–50 points (decreased happiness) and > 50 points (happiness), respectively. The mean total WHO-5 score was 58.71 ± 23.44, with a minimum value of 0 and a maximum value of 100 (Table 5).

**Table 5:** WHO-5 scores.

Items	Scores ($\overline{x}$ ± *s*)	Min	Max
Total score	58.71 ± 23.44	0	100
I have felt cheerful and in good spirits	3.20 ± 1.29	0	5
I have felt calm and relaxed	3.08 ± 1.30	0	5
I have felt active and vigorous	2.80 ± 1.42	0	5
I woke up feeling fresh and rested	2.85 ± 1.41	0	5
My daily life has been filled with things that interest me	2.75 ± 1.39	0	5

### Results of the structural equation model analysis

The hypothesized influencing factors of the PAID-5 and WHO-5 scores were analysed with a measurement model and structural model. The model could be identified, but the three paths of F2 → F5, F2 → F4, and F3 → F4 were not statistically significant. Thus, the original model was adjusted or deleted. The model was modified according to the modified index, and finally, an ideal model that was more reasonable and concise and had better adaptability was obtained. After adjustment and modification, the chi-square value of the model decreased from 1702.77 to 1060.794, the RMSEA value decreased from 0.044 to 0.032, the CFI increased from 0.829 to 0.914, and the TLI increased from 0.814 to 0.904, suggesting that the final model was better than the original model before adjustment and modification and that its fit was better.

**Figure 2:**
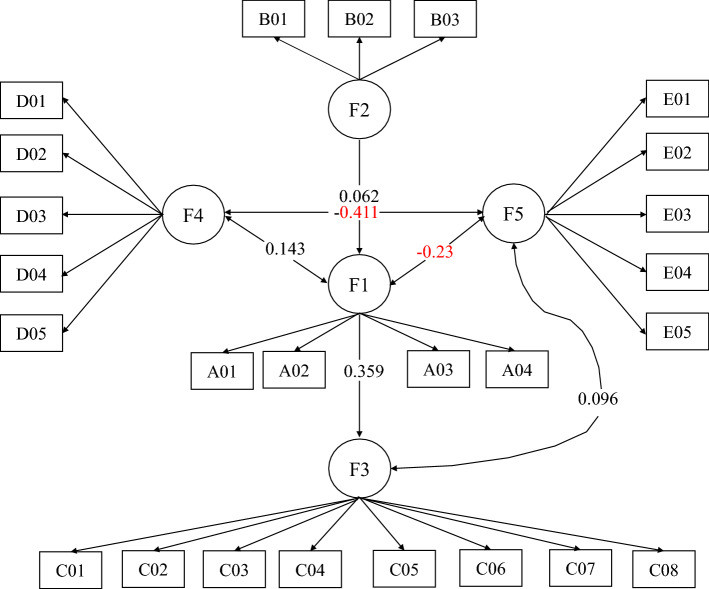
Map of the standardized paths of the structural equation model of the influencing factors of PAID-5 and WHO-5 scores.

The results of the structural equation model showed that self-management, medical history/treatment and knowledge of diabetes affected PAID-5 and WHO-5 scores (P < 0.05). In addition, exercise and economic burden had impacts on PAID-5 and WHO-5 scores (see Table 6 and Fig. 2 for the standardized path map and model parameter estimation). (1) Medical history/treatment of diabetes, knowledge and the WHO-5 score showed two-way interactive relations. Medical history/treatment of diabetes and WHO-5 exhibited a negative relation, with a coefficient of -0.23. Knowledge of diabetes and WHO-5 had a positive relation, with a coefficient of 0.096. Medical history/treatment of diabetes indirectly affected the WHO-5 score through knowledge, with an indirect effect of 0.034. (2) The medical history/treatment of DM was positively correlated with the PAID-5 score, with a coefficient of 0.143. (3) Diabetes self-management had no direct impact on PAID-5 and WHO-5 scores but indirectly affected the WHO-5 score through medical history/treatment and knowledge, with an indirect effect of 0.002; diabetes self-management also indirectly affected the PAID-5 score through medical history/treatment, with a coefficient of 0.009. (4) The correlation between the PAID-5 and WHO-5 scores was negative, with a coefficient of -0.411. It can be seen from the model that the risk factors affecting the PAID-5 and WHO-5 scores were general demographic characteristics (less exercise time and greater economic burden), medical history/treatment of diabetes (longer course of disease, more hospitalization time due to diabetes, and more acute or chronic complications), and lower levels of diabetes knowledge; in addition, solitude was also a risk factor for a low WHO-5 score.

**Table 6:** Parameter estimates of the influencing factors of emotional disorders and happiness based on the structural equation model.

Path	Parameter estimate	Statistic	*P*-value	Path	Parameter estimate	Statistic	*P-value*
F1 ← F2	0.603	7.188	0.000	C06 ← F3	0.638	22.485	0.000
F3 ← F1	0.359	6.715	0.000	C07 ← F3	0.624	19.923	0.000
F1 ↔ F4	0.143	2.527	0.012	C08 ← F3	0.558	18.579	0.000
F3 ↔ F5	0.096	2.841	0.005	D01 ← F4	0.718	–	–
F4 ↔ F5	− 0.411	− 11.046	0.000	D02 ← F4	0.732	25.691	0.000
F1 ↔ F5	− 0.23	− 4.015	0.000	D03 ← F4	0.846	22.404	0.000
A01 ← F1	0.427	–	–	D04 ← F4	0.783	21.531	0.000
A02 ← F1	0.395	7.246	0.000	D05 ← F4	0.76	20.702	0.000
A03 ← F1	0.514	8.994	0.000	E01 ← F5	0.827	–	–
A04 ← F1	0.474	7.991	0.000	E02 ← F5	0.854	25.906	0.000
B01 ← F2	0.717	-	-	E03 ← F5	0.861	22.496	0.000
B02 ← F2	0.576	7.391	0.000	E04 ← F5	0.775	19.338	0.000
B03 ← F2	0.568	7.149	0.000	E05 ← F5	0.79	19.996	0.000
C01 ← F3	0.586	–	–	F4 ← sport	− 0.086	− 3.107	0.002
C02 ← F3	0.318	12.761	0.000	F4 ← burden	0.123	4.152	0.000
C03 ← F3	0.459	16.013	0.000	F5 ← sport	0.136	5.027	0.000
C04 ← F3	0.716	22.493	0.000	F5 ← burden	− 0.081	− 2.707	0.007
C05 ← F3	0.623	18.499	0.000	F5 ← live	− 0.072	− 2.505	0.012

## Discussion

This study shows that 90.41%, 8.93% and 0.66% of subjects had diabetes-related knowledge scores of < 60 points, 60–79 points and ≥ 80 points, respectively, suggesting that the diabetes-related knowledge levels of T2DM patients is very low, which is consistent with the research results of Zhu^7^. In addition, 44.05% and 55.95% of subjects had PAID-5 scores of 0–39 (low emotional disorder) and 40–100 (severe emotional disorder), respectively, suggesting that T2DM patients generally have emotional disorders. The study confirms that the prevalence of depression, which affects the quality of life of DM patients, is higher in DM patients than in people without DM^8^. In total, 15.21%, 19.64% and 65.15% of subjects had WHO-5 scores ≤ 28 (possible depression), 29–50 (decreased happiness) and > 50 (happiness), respectively, which suggests that depression is more common in T2DM patients than in the general population. Knowledge of DM is one of the important factors that affects the self-management behaviour of DM patients^9^, and education is the most direct way to obtain knowledge. At present, the improvement of DM patients’ knowledge levels is mainly accomplished through the health education of medical staff^10^. Therefore, medical staffs still need to strengthen information sharing and education related to diabetes knowledge for T2DM patients. In addition, they should pay close attention to the moods and psychological states of DM patients, provide timely psychological counselling, strengthen effective communication, and provide comprehensive and personalized nursing and social support for long-term caregivers.

The model shows that less exercise time is a risk factor for emotional disorders and happiness. Research indicates that exercise interventions can improve individual self-efficacy, enhance self-concept, reduce stress and anxiety, and generate pleasant feelings^11^. Meanwhile, a meta-analysis also showed that exercise interventions can improve depression and anxiety in T2DM patients to a certain extent^12^. Studies have shown that with increased exercise duration (30–60 min), T2DM patients’ psychological well-being, fatigue and self-efficacy (task, time and coping) increased^13^. However, some scholars have also proposed that the regulating effect of exercise on insulin sensitivity in diabetic patients depends largely on exercise intensity rather than exercise time^14^. At present, T2DM patients are recommended to engage in high-intensity intermittent exercise and moderate-intensity continuous training. Regardless of the type of exercise, regular exercise is considered an effective means to prevent and manage T2DM and its complications and to promote individual physical and mental health.

Because of the high cost of DM treatment, the long course of the disease, and the number of hospitalizations for diabetes, the numbers of acute and chronic complications are also risk factors for emotional disorders and happiness. DM is recognized to be the third major chronic disease that threatens the health of human beings after tumours and cardiovascular and cerebrovascular disease^15^. Research shows that direct DM medical costs exceeded the growth rate of the GDP and the total national health costs in the same period^16^. In this study, 45.04% of the 1512 subjects were aged between 60 and 74 years old, and the most common occupational status was retired, with 50.79% in this category. Regarding income, 55.89% of subjects earned 1000–3000 yuan. Regarding the economic burden of medical expenses, 82.54% of these expenses were paid by individuals. In total, 67.32% of patients had DM for over 5 years. In addition, 33.6% and 60.65% had acute and chronic complication, respectively. Overall, these findings indicate that T2DM patients generally had a heavy economic burden, had long-term medical treatment, had repeated hospitalizations, covered a high proportion of their medical expenses, had low pensions or no fixed source of income, and worried about burdening their family members and placing great economic pressure to the family, resulting in a sense of self-blame and increased psychological burden. Therefore, the government and society should attach great importance to the treatment of chronic diseases such as diabetes. In the future, we should increase investment in social pension funds, improve the social security system, and expand the proportion of medical reimbursement. Furthermore, diabetic patients and families should also pay attention to blood sugar control, prevent the further development of the disease, reduce medical expenditures and reduce their economic burden.

The diabetes-related knowledge score also affected emotional disorders and happiness. The higher the diabetes-related knowledge score was, the higher the happiness index. In this study, the patients’ knowledge of diabetes was not ideal; they often had little knowledge of the disease, had a sense of uncertainty and fear, experienced a heavy psychological burden, were worried about the disease prognosis, and depended on family members and caregivers to enhance their psychological states; all of these factors contributed to patients’ emotional disorders such as depression and anxiety and decreased levels of happiness. Recently, increasing attention has been paid to health education, but the diabetes-related knowledge of clinical nurses and community nurses is generally low, and patients’ education levels and acceptance of diabetes-related knowledge also vary. The urgent task is to improve diabetes knowledge levels among nursing teams^17^ and develop individualized health education for patients so that diabetes-related knowledge can be better understood and accepted to improve the effectiveness of treatment activities.

Solitude is a risk factor for low levels of happiness in T2DM patients. The family is the best source of emotional support for patients. Effective family support can help patients obtain psychological and emotional support and reduce their psychological burden. La et al.^18^ compared family support with support from friends, and family support had more influence on blood glucose monitoring, insulin injection, diet and exercise. Some studies have shown that the higher the level of family support, the lower one’s incidence of depression is, and the more stable one’s psychological state is^19^. Good family support can help patients reduce the pain of disease, increase confidence in life, and improve quality of life. Therefore, it is recommended that diabetic patients live with their families as much as possible, encourage their families to accompany them and communicate with them more, enjoy family warmth and support, and improve their levels of happiness.

In conclusion, a structural equation model was used to explore the influence of self-management, medical history/treatment and knowledge on emotion and happiness, as well as the direct and indirect effects of influencing factors on emotional disorders and happiness. At present, the diabetes-related knowledge of T2DM patients is very low, and these patients generally experience emotional disorders. Medical teams should not only develop personalized health education to allow diabetic patients to obtain relevant knowledge but also create a good social health governance environment, pay more attention to patients' emotions, and work with society and families to ensure that DM patients have enough family support and social support.

## Data Availability

The datasets used and/or analysed during the current study available from the corresponding author on reasonable request.
